# Cubic gauche polymeric nitrogen under ambient conditions

**DOI:** 10.1038/s41467-017-01083-5

**Published:** 2017-10-13

**Authors:** El Mostafa Benchafia, Zhenhua Yao, Gu Yuan, Tsengmin Chou, Hong Piao, Xianqin Wang, Zafar Iqbal

**Affiliations:** 1 Interdisciplinary Program in Materials Science and Engineering, New Jersey Institute of Technology, Newark, NJ 07102 USA; 2 Department of Chemical, Biological and Pharmaceutical Engineering, New Jersey Institute of Technology, Newark, NJ 07102 USA; 3 Laboratory for Multiscale Imaging, Stevens Institute of Technology, Hoboken, NJ 07030 USA; 4 Chemistry and Chemical Engineering, General Electric Co., Niskayuna, NY 12309 USA; 5 Department of Chemistry and Environmental Science, New Jersey Institute of Technology, Newark, NJ 07102 USA

## Abstract

The long-sought cubic gauche phase of polymeric nitrogen (cg-PN) with nitrogen-nitrogen single bonds has been synthesized together with a related phase by a radio-frequency plasma reaction under near-ambient conditions. Here, we report the synthesis of polymeric nitrogen using a mixture of nitrogen and argon flowing over bulk β-sodium azide or β-sodium azide dispersed on 100 nm long multiwall carbon nanotubes. The cg-PN phase is identified by Raman and attenuated total reflection-Fourier transform infrared spectroscopy, and powder X-ray diffraction. The synthesis of the cubic gauche allotrope of high energy density polymeric nitrogen under near-ambient conditions should therefore enable its optimized production and applications as a “green” energetic material and a potential catalyst for different chemical reactions.

## Abstract (web summary)

Polymeric phases of nitrogen are promising as environmentally-friendly, high energy-density materials, but are inherently unstable. Here, the authors report the synthesis and stabilization of polymeric nitrogen in its cubic gauche phase under near-ambient conditions, via plasma-enhanced chemical vapour deposition.

## Introduction

A large amount of energy (2.3 eV per atom) is expected to be released upon transforming singly-bonded nitrogen to diatomic triply-bonded molecular nitrogen. This chemical energy can be ideally stored during the transformation of a triple bond to three single bonds in polymeric nitrogen to form a high energy density material^1^. Consequently, single-bonded polymeric nitrogen should be a high energy density material with a range of applications as a metal-free “green” energetic material, or as a propellant. The N_3_^−^ azide anion has been known since 1890, but it was not until a hundred years later that the all-nitrogen N_5_^+^ cationic species stabilized in the N_5_^+^AsF_6_^−^ salt was synthesized^2^. Single-bonded polymeric nitrogen in the cubic gauche phase (cg-PN) was predicted by Mailhiot et al.^3^, and was first produced in extremely small quantities in a diamond anvil pressure cell by Eremets and coworkers^4, 5^ from molecular nitrogen under extreme conditions of pressure and temperature (210 GPa and 2000 K). The polymeric nitrogen phase thus formed rapidly dissociated back to nitrogen under ambient conditions. Prior to this work, Eremets and coworkers^6^ synthesized an extended, non-molecular semiconducting nitrogen phase at 300 K and 240 GPa that is stable at ambient pressure below 100 K. This structure is amorphous, based on its broad Raman and infrared spectral features. More recently, Hirshberg et al.^7^ theoretically predicted another nitrogen-based solid, an N_8_ molecular crystal that is stable at ambient pressure.

Earlier investigations to obtain non-molecular polymeric phases of nitrogen at lower pressures up to 160 GPa and temperatures from 120 K to 3300 K, were also carried out by Eremets et al.^8^ and by Popov^9^ using β-sodium azide (NaN_3_) instead of molecular nitrogen as the precursor. A so-called phase I was observed at 19 GPa by Eremets et al.^8^ and proposed to have an azide ion intercalated nitrogen network structure based on its Raman spectrum. At 50 GPa, another phase referred to as phase II was observed with a fully extended nitrogen structure. In contrast to Eremets et al.^8^, Popov^9^ attributed phase II to the cg-PN phase based on the appearance of two Raman lines near 785 cm^−1^ and 1140 cm^−1^ at 50 GPa, which can be attributed to the *A* and *E* symmetry modes of cg-PN predicted by the density functional perturbation theory calculations of Caracas^10^.

Here, we describe the synthesis and stabilization of polymeric nitrogen in its cubic gauche phase under near-ambient conditions employing plasma-enhanced chemical vapour deposition which is known to form high pressure phases, such as that of diamond at ambient conditions. Most of the work involved the use of sodium azide as the precursor for the synthesis, with and without carbon nanotubes as the substrate. A few experiments were also carried out with nitrogen gas as precursor on carbon nanotube substrates. Characterization was performed using Raman and attenuated total reflectance (ATR)-Fourier transform infrared (FTIR) spectroscopy, powder X-ray diffraction, high resolution transmission electron microscopy and selected area electron diffraction at −165^o^ C. In addition, X-ray photoelectron spectroscopy (XPS) and temperature programmed desorption (TPD), discussed in Supplementary Note 2, were conducted to rule out the likelihood of pyrrolic nitrogen formation and confirm the singly N-N bonded polymeric phase.

## Results

### Raman and ATR-FTIR spectroscopy

A number of samples with the same Raman spectral features were synthesized by radio-frequency plasma-induced reactions of sodium azide supported on short carbon nanotubes and on unsupported sodium azide powder. Carbon nanotubes are chosen as the substrate since Abou-Rachid et al.^11^ proposed its confining and stabilizing function for metastable N_8_ polymeric nitrogen clusters. However, our experimental results show that a carbon nanotube substrate is not a requirement for cg-PN formation.

**Fig. 1:**
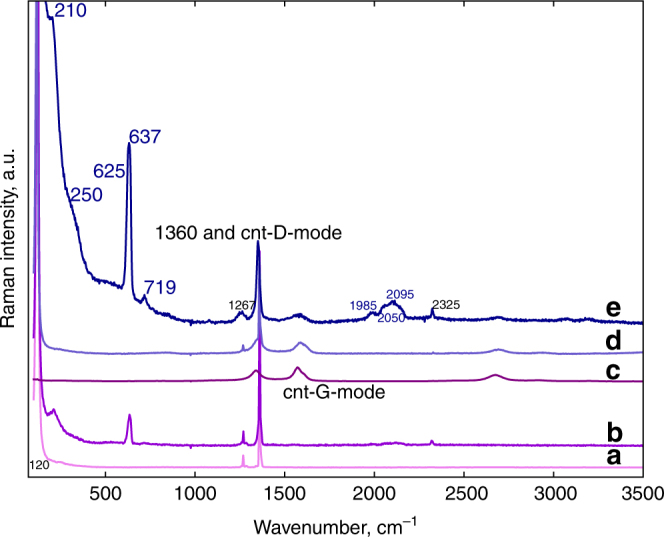
Raman spectra (532 nm) of the samples before and after plasma reaction. Spectra **a**, **b** are from free-standing NaN_3_ before and after plasma reaction, respectively; spectra **d**, **e** are from NaN_3_ deposited from sodium azide solution on short carbon nanotubes before and after plasma reaction, respectively, and spectrum **c** is from as-received short carbon nanotubes. NaN_3_ has three Raman active modes: the symmetric stretching ν_1_ mode of the azide ion at 1360 cm^−1^, the librational lattice mode at 120 cm^−1^ and the first overtone of the IR active bending ν_2_ mode of the azide ion at 1267 cm^−1^. The 1360 cm^−1^ line from the azide ion overlaps with the carbon nanotube D (defect) mode. The carbon nanotube G (graphitic) mode is at 1585 cm^−1^, the sharp line at 2325 cm^−1^ in the plasma-reacted samples may be attributed to nitrogen trapped in the short nanotubes and in sodium azide, and the line at 2620 cm^−1^ is the carbon nanotube 2D mode

Raman spectra obtained from free-standing, unreacted and plasma-reacted sodium azide, and from sodium azide deposited on short multiwall carbon nanotube sheets before and after plasma reaction, are shown in Fig. 1. After plasma reaction of sodium azide on carbon nanotubes the following new lines are observed: A low frequency line at 210 cm^−1^ with a shoulder at 250 cm^−1^, a weak, broad feature near 500 cm^−1^, an intense line at 637 cm^−1^ with a weaker shoulder near 625 cm^−1^, a new line at 719 cm^−1^ with a factor of 10 lower intensity than the line at 637 cm^−1^, and a low intensity broad feature with peaks at 1985, 2050 and 2095 cm^−1^. In addition, the region around 1360 cm^−1^ consists of two lines associated with unreacted sodium azide at 1267 cm^−1^ and 1360 cm^−1^ assigned to the 2ν_2_ and ν_1_ modes of the azide ion, respectively. The other features in the Raman spectrum consist of a line at 1365 cm^−1^ due to the D (defect) mode of the carbon nanotube substrate together with lines at 1585 cm^−1^ and near 2630 cm^−1^ due to the nanotube G (graphitic) and 2D modes, respectively, of the carbon nanotube substrate. Similar features except for the absence of the lines due to the nanotubes are observed for plasma-reacted free-standing sodium azide in Fig. 1b.

The emergent, intense line at 637 cm^−1^ and the much weaker line at 719 cm^−1^ can be definitively assigned to the pore breathing *A* symmetry and N-N tilting *T(TO)* symmetry Raman-active modes, respectively, of cg-PN extrapolated to ambient pressure according to the calculation of Caracas^10^. These lines correspond to the intense line at 830 cm^−1^ and the factor of 100 weaker doublet at 963 cm^−1^ observed at 110 GPa in the diamond anvil cell by Eremets and coworkers^4, 5^ for the cg-PN phase. The density functional theory calculations of the peak positions and intensities for the Raman-active modes at zero pressure carried out for the cg-PN phase (shown in Supplementary Fig. 2) are in agreement with the calculation of Caracas^10^ and show that the *A* symmetry line at 637 cm^−1^ is the predominant peak in the Raman spectrum of cg-PN at about zero pressure. The possibility that the intense line at 637 cm^−1^ is due to azide defects created by chemical reactions on the edges of the short nanotubes is ruled out on the basis of experiments on the reaction of sodium azide solutions with short nanotubes (see Supplementary Fig. 5 and discussion in Supplementary Note 1). The Raman spectra in Supplementary Fig. 5 for these reacted short nanotubes clearly show the absence of Raman lines around 630 cm^−1^ – the infrared results are discussed below. The substantially increased intensities of the 637 cm^−1^ and 719 cm^−1^ lines in Fig. 1e for plasma-reacted sodium azide on carbon nanotubes can be attributed to cg-PN stabilized by the carbon nanotube walls as shown theoretically for N_8_ oligomers related to cg-PN by Abou-Rachid et al.^11^ and experimentally for N_8_^−^ anions synthesized by electrochemical cyclic voltammetry by Wu et al.^12^, respectively.

The new Raman lines at 210, 250, 1985, 2050 and 2095 cm^−1^, and a weak, broad feature at 500 cm^−1^ (indicated by an arrow in Fig. 1) together with the shoulder at 625 cm^−1^ noted above can be assigned to the ambient pressure stabilized phase I observed in high pressure/high temperature treated sodium azide by Eremets et al.^8^ and Popov^9^. The lines at 210, 250 and 1985 cm^−1^ can be assigned to vibrations of the nitrogen network in phase I, the features at 500, 625, and 1267 cm^−1^ are assigned to Raman-activated ν_2_ and 2ν_2_ (part of the intensity of this overtone would also come from unreacted azide ions present in the sample), whereas the line at 2095 cm^−1^ can be assigned to the Raman-activated ν_3_ vibration of the azide ions intercalated in the phase I structure. The structure of this phase, based on the qualitative model proposed by Eremets et al.^8^, is that of an extended nitrogen structure with intercalated azide ions, where interaction of the azide ions with the extended nitrogen network results in activation of the Raman-inactive bending (ν_2_) and asymmetric stretching (ν_3_) modes of the azide ion.

**Fig. 2:**
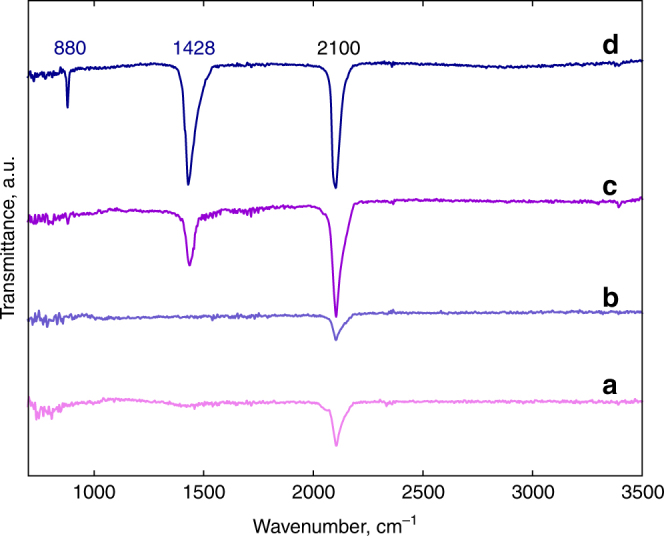
ATR-FTIR spectra of the samples before and after plasma reaction. Spectra **a**, **c** are taken from pure NaN_3_ without carbon nanotube support before and after plasma reaction, respectively; and spectra **b**, **d** are from plasma-reacted NaN_3_ deposited on short MWNTs before and after plasma reaction, respectively. The IR-active modes for NaN_3_ are the bending ν_2_ mode of the azide ion at 638 cm^−1^ (not shown here) and the anti-symmetric stretching ν_3_ mode of the azide ion at 2100 cm^−1^

ATR-FTIR spectra of samples produced by plasma reaction from free-standing bulk sodium azide and from sodium azide deposited on short multiwall carbon nanotubes are depicted in Fig. 2. New lines due to extended nitrogen phases occur after plasma reaction at 1428 cm^−1^ and 880 cm^−1^, whereas the line at 2100 cm^−1^ is the infrared active ν_3_ line of unreacted azide ions. The line with lower intensity at 880 cm^−1^ can be clearly assigned to the *T(TO)* symmetry vibration of cg-PN extrapolated to near zero pressure in agreement with the calculation of Caracas^10^. The relatively strong and broader line at 1428 cm^−1^, however, does not correspond to any of the infrared-active phonon frequencies of cg-PN computed either by Caracas^10^ or in the present work (Supplementary Fig. 2). On the other hand, a line at 1550 cm^−1^ together with a relatively sharp feature at 750 cm^−1^ is observed by synchrotron infrared spectroscopy in nitrogen compressed to 134–160 GPa in a diamond anvil pressure cell by Gornachov et al.^13^. This line can be extrapolated to the 1400 cm^−1^ region at ambient pressure and together with the presence of the line at 750 cm^−1^ would suggest that the phase involved in the Gornachov et al.^13^ work is phase I from sodium azide, where modified azide ions are intercalated into a nitrogen network. The line at 1428 cm^−1^ can therefore be tentatively assigned to a modified stretching mode of the azide ions in phase I. Moreover, these lines do not appear in the ATR-FTIR spectrum of short MWCNTs shown in Supplementary Fig. 4, ruling out the formation of defects on the edges of the nanotubes by interaction with the azide solution.

Comparing the ATR-FTIR spectra in Fig. 2d and c from plasma-reacted sodium azide with and without carbon nanotubes, respectively, it is observed that the lines at 880 cm^−1^ and 1428 cm^−1^ occur in both instances but with increased intensity when carbon nanotubes are used as the substrate. Our results therefore demonstrate that cg-PN can be synthesized even without a carbon nanotube substrate. However, more cg-PN are present with the carbon nanotube substrate possibly due to the more effective distribution of the precursor on the high surface area of the carbon nanotubes or stabilization on the tube sidewalls, as proposed by Abou-Rachid et al.^11^ According to this theoretical calculation^11^, the first conduction band of the N_8_ chain interacts with the conduction band of the nanotube resulting in chain-nanotube hybridization and ultimately to partial charge transfer leading to a net positive charge on the inner wall of the nanotube and a net negative charge on the N_8_ chains that stabilizes the nitrogen chains. Similarly, positively charged channels within the rhombohedral structure of β-sodium azide with alternating layers of sodium and azide ions parallel to the (111) direction, formed during the early stages of plasma reaction can function in the same manner to stabilize negatively charged nitrogen oligomer chains in the structure. In both cases, the cg-PN structure comprised of these N_8_ clusters is formed. On the other hand, as noted for the Raman and infrared results above, it is likely that the plasma reaction is more effective for the highly dispersed sodium azide on the nanotubes than in the bulk, free standing azide, and therefore larger amounts of the extended nitrogen structures are produced on the nanotubes. Moreover, the low effective kinetic temperature of the particles in a plasma system is likely to allow crystallization of unusual and perhaps metastable phases^14^.

### X-ray powder diffraction and transmission electron microscopy

**Fig. 3:**
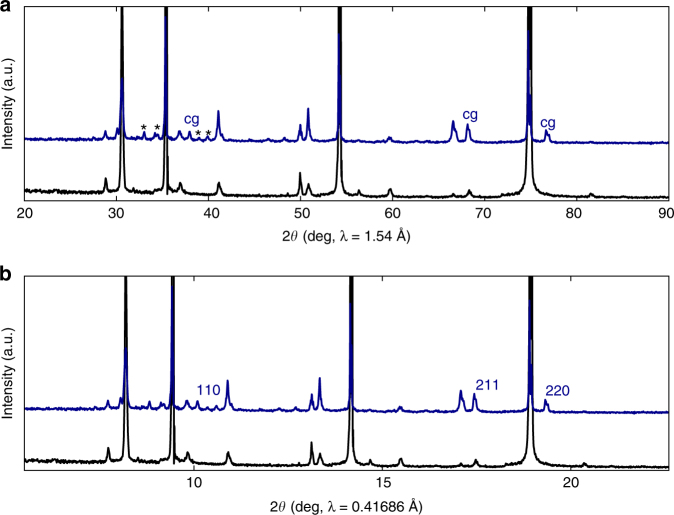
X-ray powder diffraction patterns. **a** Diffraction patterns measured using CuKα radiation for NaN_3_ powder before (diffraction pattern at the bottom) plasma reaction and after (diffraction pattern on top) plasma reaction. The lines indicated by < cg > at 37, 66 and 77 2θ degrees correspond closely to the (110), (211) and (220) reflections of the cubic gauche structure of polymeric nitrogen reported by Eremets and co-workers^4, 5^. The strong lines correspond to unreacted, pristine NaN_3_, and the lines indicated by asterisks can be from phase I discussed in the text or a sodium nitride phase. **b** The X-ray diffraction pattern converted to the X-ray beam wavelength of 0.41686 Å used by Eremets and coworkers^4, 5^. The (110), (211) and (220) reflections of the cubic gauche structure emerge clearly in the plasma-reacted sample (top) and are completely absent in the pristine NaN_3_ sample (bottom)

X-ray diffraction measurements were carried out on the samples from the plasma-reacted free-standing sodium azide in order to avoid any interference from the carbon nanotube substrates. Diffraction patterns from sodium azide before and after the plasma-reaction are displayed in Fig. 3. The top panel shows the data taken with λ ~ 1.54 Å and the bottom panel shows the same diffraction pattern converted to that for λ = 0.41686 Å employed by Eremets et al.^4, 5^, using the Rietveld FULLPROF suite program. Reflections due to cubic gauche polymeric nitrogen clearly emerge as indicated in the top panel, and the associated indices are shown on the bottom panel. Lines shown with asterisks are likely to arise from a sodium nitride phase or from the PN phase I discussed above. The (200) line near the 2θ value of 13.5^o^ on the bottom panel and at about 41.0^o^ on the top panel overlaps with a sodium azide reflection that shows a substantial enhancement in the plasma-reacted sample due to the (200) reflection from the cg-PN structure. The new reflections are approximately 10 volume% of the sample and the remaining component is from unreacted sodium azide. It is important to note here that the reflections assigned to the cubic gauche phase are being compared to data obtained at high pressures and temperatures by Eremets et al.^5^. However, because of the extremely high modulus for this material determined by Eremets et al.^5^ the lattice spacings are not expected to change by much under ambient conditions.

**Fig. 4:**
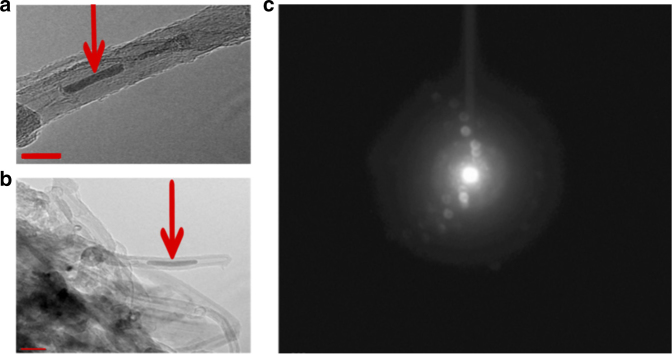
High resolution TEM images. Panels **a**, **b** show a solid structure on the short multiwall carbon nanotubes (indicated by arrows) obtained at −165^o^C. The scale marker (bottom left) in panel **a** is 10 nm and the scale marker (bottom left) in panel **b** is 20 nm. The selected area electron diffraction pattern is shown in panel **c**. The inner ring is from the carbon nanotubes and the second ring is from the synthesized phase which is highly crystalline. Analysis of the electron diffraction pattern using FFT (Fast Fourier Transform) is displayed in Supplementary Fig. 3 from selected regions of the image in panel **a**

TEM images and selected area electron diffraction taken at −165^o^C to minimize thermal decomposition from a plasma-reacted sample of sodium azide deposited on a short carbon nanotube substrate, are shown in Fig. 4. The images show the formation of a solid structure inside the central tube of the MWCNTs in Fig. 4a from which a crystalline selected area electron diffraction pattern is obtained (further details are provided in the figure caption and in the Supplementary Information). The FFT (Fast Fourier Transform) pattern in Supplementary Fig. 3 from the diffraction pattern in Fig. 4c shows the appearance of a new spot with a lattice spacing at about 2.050 Å together with reflections from the carbon nanotube substrate. The spacing near 2.050 Å is 0.39 Å lower than the (110) spacing and 0.32 Å higher than the (200) spacing reported for cg-PN by Eremets et al.^5^. It also does not correspond to any of the lattice spacings for β- and α- sodium azide or leftover catalyst in the nanotubes, in agreement with the absence of sodium and other metals in energy dispersive X-ray spectroscopy (EDX) data taken both in TEM and by low energy scanning electron microscopy.

### X-ray photoelectron spectroscopy and temperature-programmed desorption

Core level N(1 s) X-ray photoelectron spectroscopy (XPS) is sensitive to nitrogen as shown by the spectra (Supplementary Fig. 6) from sodium azide modified MWCNT, nitrogen plasma-reacted on MWCNT, and reference unreacted samples, sodium azide and carbon nitride. The azide modified MWCNT sample was prepared by UV-irradiation of azide-soaked MWCNT. The plasma-reacted sample was prepared on carbon nanotube substrates in a nitrogen gas environment instead of sodium azide in order to eliminate contributions due to unreacted sodium azide in the XPS spectrum. The azide-modified and plasma-reacted samples were compared to the pure sodium azide and carbon nitride reference samples. The signals from both azide-modified and plasma-reacted samples were too weak to be observed without multiplying the signals by a factor of 10^4^. The peaks from the azide-modified sample are similar in energy but not in intensity ratio compared to the spectrum from pure sodium azide. The higher intensity of the line near 400 eV and its shift slightly to lower binding energy compared to that for pure sodium azide may indicate some amorphous PN formation as discussed below for plasma-reacted nitrogen on nanotubes. The weak feature at ~ 404 eV is probably from a species bonded to the carbon nanotubes. This line near 400 eV is however unlikely to be due to pyrrolic nitrogen since the TPD (temperature-programmed desorption) scan (Supplementary Fig. 7) from this sample did not show a high temperature decomposition peak that would be expected for pyrrolic nitrogen. These features from the azide-modified and reference samples are entirely different from the N1s XPS spectrum from the plasma-reacted nitrogen sample which has primarily one feature at ~ 398 eV consistent with that of the singly N-N bonded cg-PN phase. This result shows that a PN phase can be obtained by plasma reaction with both nitrogen and sodium azide as precursors, but for plasma-reacted nitrogen only small fractions of PN are produced which preliminary selected area electron diffraction indicates to be largely amorphous.

The material in the central tube of the MWCNTs in Fig. 4a and b may be attributed to the intermediate phase I PN structure reported by Eremets et al.^8^ based on the Raman and infrared spectra discussed above but a more definitive assignment via electron diffraction is not possible since the lattice parameters for the phase I structure are presently unknown. The central tube of the MWCNTs has a diameter of 5–6 nm (Fig. 4a and b) whereas the particle size uncorrected for strain from the X-ray diffraction data for the new lines in Fig. 3, determined from Scherrer’s formula using a rod-like shape factor of 0.6, ranges from 8 to 13 nm. This would provide a tight fit inside the central tube that could explain some of the bulging of the nanotube sidewalls evident, for example, in the image in Fig. 4b.

## Discussion

Radio frequency plasma-enhanced chemical vapour deposition using sodium azide as precursor, with and without carbon nanotube substrates, and nitrogen and argon as carrier gases, has produced the long-sought cg-PN phase under ambient conditions, co-existing with the so-called phase I structure reported by Eremets et al.^8^ Strong evidence for cg-PN is provided by powder X-ray diffraction data taken on plasma-reacted pure sodium azide, and the appearance of the intense *A* symmetry breathing mode Raman line of cg-PN at 637 cm^−1^ extrapolated to ambient pressure in agreement with the calculation of Caracas^10^, and by ATR-FTIR spectra, for both plasma-reacted pure sodium azide and sodium azide dispersed on short carbon nanotubes. Evidence for a crystalline structure on MWCNTs was also obtained by high resolution transmission electron microscopy and selected area electron diffraction at −165^o^C with a lattice spacing of 2.050 Å, which does not correspond to the lattice spacings for cg-PN or those of the β or α phases of sodium azide. It could however correspond to a PN phase I for which the crystal structure is unknown. The strong evidence for a successful synthesis of the cubic gauche allotrope of high energy density polymeric nitrogen under ambient conditions would therefore make it possible to explore its applications as a high energy density energetic material and as a potential catalytic material for many chemical reaction systems^12^. X-ray photoelectron spectroscopy and temperature programmed desorption on PN and related reference samples were also used to identify the type of the nitrogen present.

## Methods

### Sample preparation

Short multiwall carbon nanotubes were obtained from Cheap Tubes Inc. (Brattleboro, VT). The as-received nanotubes used are about 50–100 nm in length and about 10 nm in diameter. They were intentionally chosen to be short in length to allow for the nitrogen polymer to form inside the tubes. The carbon nanotubes were mixed with sodium dodecyl sulfate (SDS) surfactant followed by horn sonication and a vacuum filtration process to produce free-standing nanopaper substrates. The nanopaper substrates were then dipped in sodium azide in a pH = 4 aqueous buffer solution overnight and then dried in air for a few hours.

### The plasma process

The schematic of the plasma apparatus used for the synthesis is depicted in Supplementary Fig. 1. Nitrogen and argon were fed into the quartz tube through needle valve flow meters allowing control of the flow rate of the gas precursors. An adjustable radio frequency generator delivering up to 500 W is attached to an adjustable impedance matching box. Throughout this study, a power range of 65–100 W was maintained. Gas mixtures of 50% nitrogen and 50% argon were introduced into the deposition chamber where a quartz boat was used to hold a 2 × 2 cm^2^ nanopaper substrate. A vacuum pump was used to evacuate the deposition chamber to pressures below 1 Torr. Flow rates of 10–15 sccm (standard cubic centimetres per minute) were maintained for each gas. The deposition time was typically between 2 and 3 h, and at least 2 h was required for optimal synthesis. The temperature of the substrate throughout each experiment was monitored using a thermocouple even though external heat was not applied to the reactor. Temperatures of the plasma jet were in the 200–300 °C range and monitored using a thermocouple under floating potential inside the quartz tube.

### Raman, ATR-FTIR, XRD and TEM

Raman spectroscopy was carried out with a Thermo Scientific DXR micro-Raman spectrometer (Thermo Scientific, Waltham, MA, USA) with 532-nm laser excitation at a spectral resolution of 2 cm^−1^ and a spatial resolution of 10 μm. Fourier transform infrared (FTIR) spectroscopy was conducted using a Magna Model 560 instrument (Nicolet Instrument Corporation, Madison, WI, USA) attached to an attenuated total reflectance (ATR) accessory with a single reflection ZnSe crystal (Pike Technologies, Madison, WI, USA). X-ray diffraction (XRD) measurements were carried out on a Philips PW3040 X-ray Diffractometer in the range from 10° to 90° with CuKα radiation (λ = 1.54 Å) with a step size of 0.02°.

TEM imaging and selected area electron diffraction were conducted using a Philips field emission CM20 TEM/STEM system coupled to a low temperature sampling attachment with a Gatan 792 Multi-scan CCD (1 K × 1 K) camera, Schottky field emitter and TWIN pole pieces, objective lens: 0.282 nm point resolution, 0.144 nm line resolution and 0.180 nm information limit at 200 KV (TEM mode).

### Theoretical calculations

Density functional theory (DFT) calculations were carried out using the Quantum Espresso^15^ package with the local density approximation (LDA) using the Von Barth-Car norm-conserving pseudopotential with wave function cutoff energies of 120 Ry and a 12 × 12 × 12 q-point mesh for the first Brillouin zone.

### XPS and TPD

The XPS data was collected using a Kratos Axis Ultra DLD spectrometer. The source is monochromatic Al Kα radiation (1486.6 eV) operating at 225 W. The spectra were collected using the combination of electrostatic and magnetic lens (hybrid mode) for large area acquisition (700 by 300 mm). Pass energies of 160 and 20 eV were used for survey and high-resolution scans, respectively. All the data analysis was accomplished using the commercial software Casa XPS, version 2.2.14, supplied by Casa Software Ltd. Temperature-programmed desorption (TPD) was carried out using a Micromeritics® AutoChem II 2920 system.

### Data availability

The data that support the findings of this study are available within the article and Supplementary Information Files. The data are also available from the corresponding authors upon request.

## Electronic supplementary material

Supplementary Information

## References

[1] Eremets, M. I., Trojan, I. A., Gavriliuk, A. G. & Medvedev, S. A. in *Static Compression of Energetic Materials* (eds Peiris, S. M. & Piermarini, G. J.) (Springer Berlin Heidelberg, 2008).

[2] Christe KO, Wilson WW, Sheehy JA, Boatz JA (1999). N_5_^+^: a novel homoleptic polynitrogen ion as a high energy density material. Angew. Chem. Int. Ed..

[3] Mailhiot C, Yang LH, McMahan AK (1992). Polymeric nitrogen. Phys. Rev. B.

[4] Eremets MI, Gavriliuk AG, Trojan IA, Dzivenko DA, Boehler R (2004). Single-bonded cubic form of nitrogen. Nat. Mater..

[5] Trojan IA, Eremets MI, Medvedev SA, Gavriliuk AG, Prakapenka VB (2008). Transformation from molecular to polymeric nitrogen at high pressures and temperatures: *In situ* x-ray diffraction study. Appl. Phys. Lett..

[6] Eremets MI, Hemley RJ, Mao H, Gregoryanz E (2001). Semiconducting non-molecular nitrogen up to 240 GPa and its low-pressure stability. Nature..

[7] Hirshberg B, Gerber RB, Krylov AI (2014). Calculations predict a stable molecular crystal of N_8_. Nat. Chem.

[8] Eremets MI (2004). Polymerization of nitrogen in sodium azide. J. Chem. Phys..

[9] Popov M (2005). Raman and IR study of high-pressure atomic phase of nitrogen. Phys. Lett. A..

[10] Caracas R (2007). Raman spectra and lattice dynamics of cubic gauche nitrogen. J. Chem. Phys..

[11] Abou-Rachid H, Hu A, Timoshevskii V, Song Y, Lussier L-S (2008). Nanoscale high energetic materials: a polymeric nitrogen chain N_8_ confined inside a carbon nanotube. Phys. Rev. Lett..

[12] Wu Z, Benchafia EM, Iqbal Z, Wang X (2014). N_8_^-^ polynitrogen stabilized on multi-wall carbon nanotubes for oxygen-reduction reactions at ambient conditions. Angew. Chem. Int. Ed..

[13] Goncharov AF, Gregoryanz E, Mao H-K, Liu Z, Hemley RJ (2000). Optical evidence for a nonmolecular phase of nitrogen above 150 GPa. Phys. Rev. Lett..

[14] Vajenine GV (2008). On reactions between alkali metals and active nitrogen. Solid State Physics.

[15] Giannozzi P (2009). QUANTUM ESPRESSO: a modular and open-source software project for quantum simulations of materials. J. Phys.: Cond. Matt.

