# Supplementary material for: Cubic gauche polymeric nitrogen under ambient conditions
Source: Nat Commun. 2017 Oct 13;8:930. doi: 10.1038/s41467-017-01083-5 (PMC5640604; doi:10.1038/s41467-017-01083-5)
Supplement: Supplementary file 1 — Supplementary Information [file 41467_2017_1083_MOESM1_ESM.pdf]

**Supplementary Figure 1.** Schematic drawing of the plasma system used in this study.

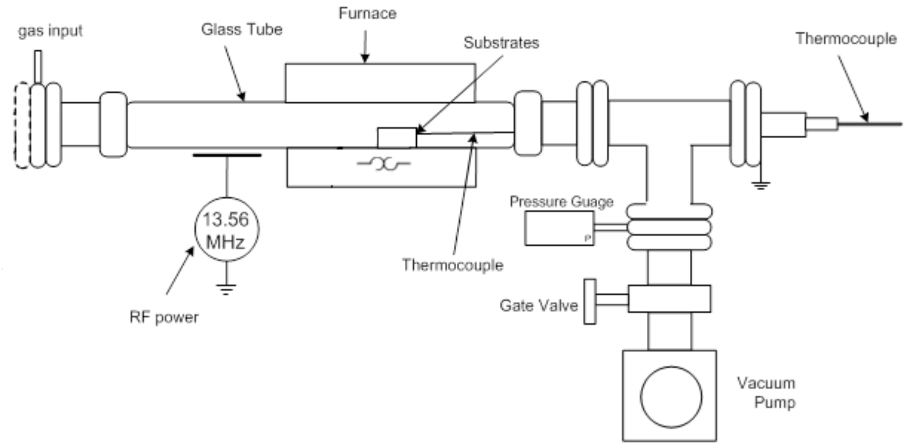

**Supplementary Figure 2.** The calculated Raman and IR spectra of cg-PN at zero pressure. The Raman spectrum is dominated by the A symmetry mode and the IR activity is relatively weak. The intensity of the A mode is 76.7754 Å$^4$/amu and is truncated to show the remaining lines. The local density approximation (LDA) approach has a tendency to underestimate the force constants and vibrational frequencies because it underestimates bond lengths and angles.

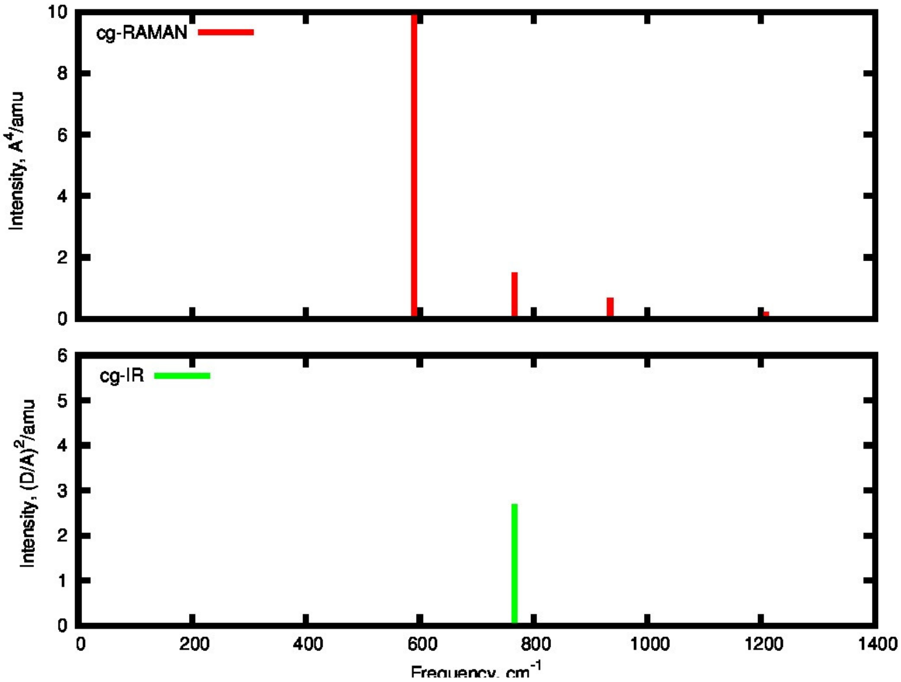

**Supplementary Figure 3.** Fast Fourier Transform (FFT) analysis of the selected area electron diffraction pattern taken from TEM image regions indicated in the top panel (The scale marker is 10 nm). The spacing obtained from the unknown region is discussed in the main text of the paper.

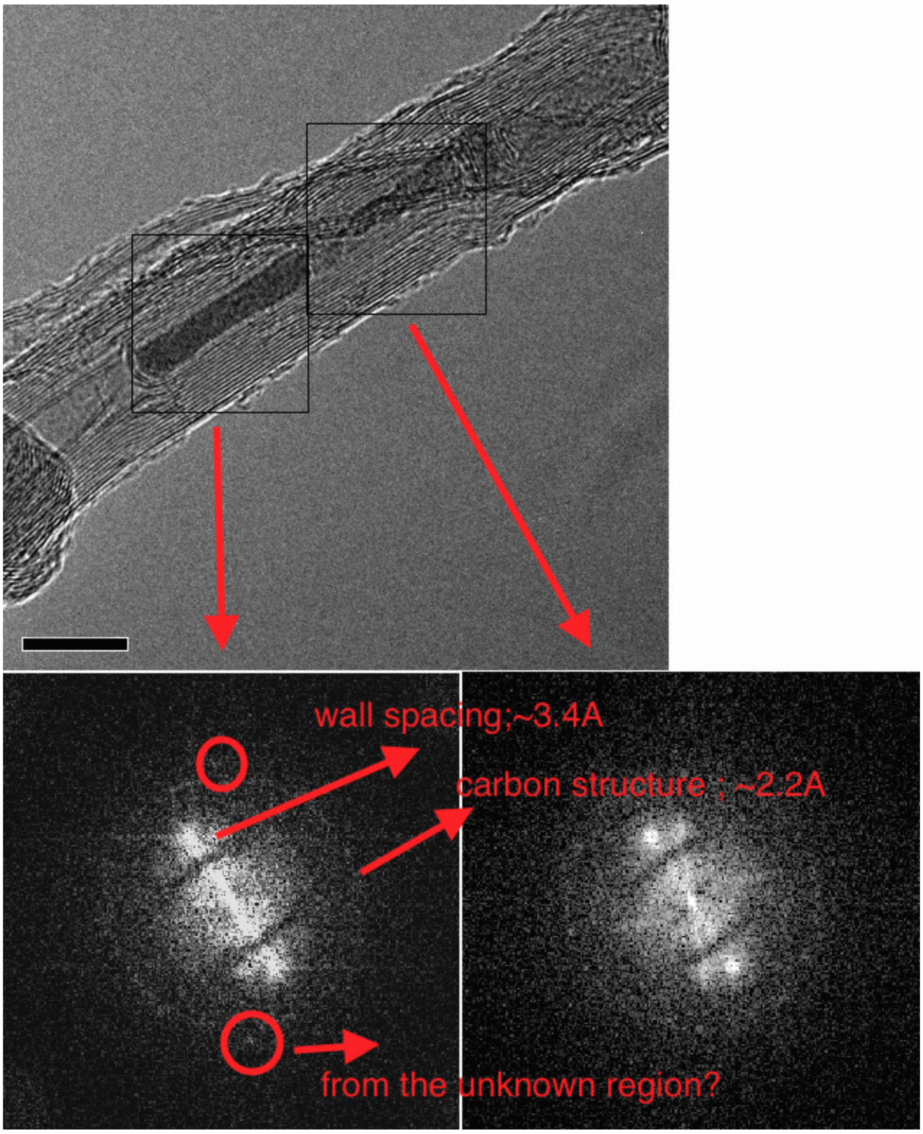

**Supplementary Figure 4.** ATR-FTIR spectra of as-received short MWCNTs (blue), as-received long, conventional MWCNTs (black), sodium azide solution soaked short MWCNTs (green) and sodium azide soaked long, conventional MWCNTs (red). The line at 2100 $cm^{-1}$ is the $\nu_3$ asymmetric stretching mode of the azide ion.

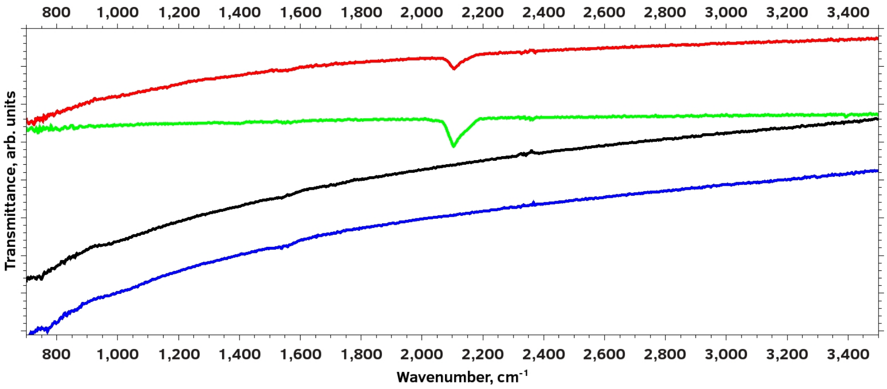

**Supplementary Figure 5.** Raman spectra of as-received short MWCNTs (blue), as-received long, conventional MWCNTs (black), sodium azide soaked short MWCNTs (green) and sodium azide soaked long, conventional MWCNTs (red). The sharp line at 1360 $cm^{-1}$ is the $\nu_1$ Raman-active symmetric stretching mode of the azide ion.

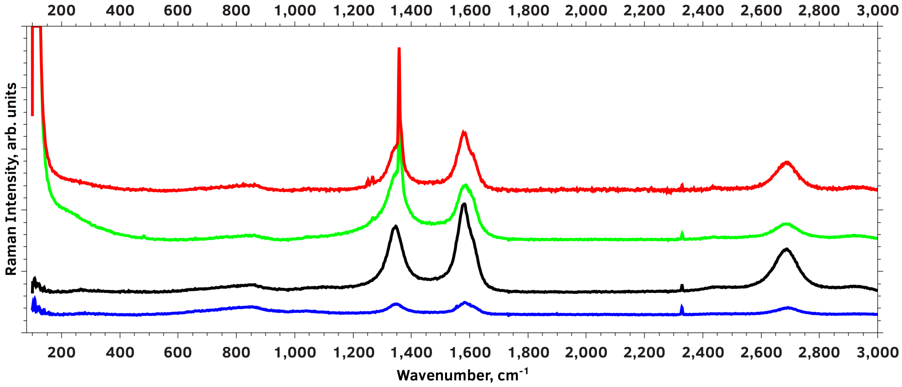

**Supplementary Figure 6.** N1s XPS spectra from sodium azide modified MWCNT (green curve), nitrogen plasma-reacted on MWCNT (blue curve), and reference unreacted samples, sodium azide (red curve) and carbon nitride (black curve).

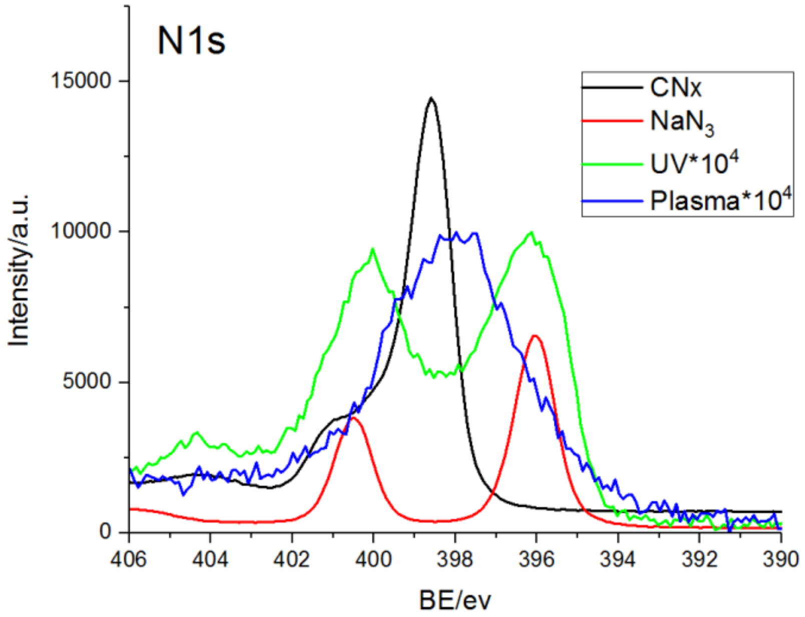

**Supplementary Figure 7.** Temperature-programmed desorption (TPD) profiles of unreacted carbon nitride (red), azide modified MWNT (green), and unreacted sodium azide (black).

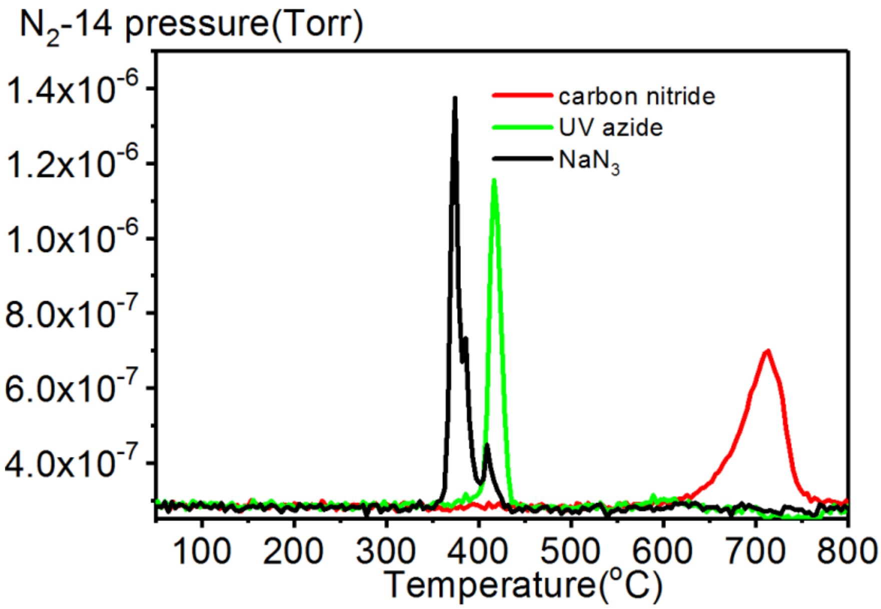

**Supplementary Note 1: Carbon nantubes interaction with sodium azide.** Sodium azide was interacted with the nanotubes by soaking MWCNTs in sodium azide solution until the MWCNTs were fully saturated with $N_3^-$ species. Comparing both the FTIR and Raman spectra, we can see that $N_3^-$ interacted short (as well as the long) MWCNTs do not have an FTIR peak at 880 $cm^{-1}$ and a Raman peak at 637 $cm^{-1}$ which are assigned to cg-PN. This confirms that these lines appear on plasma reaction of sodium azide to form cg-PN, and are not due to chemisorbed azide ion species on the short MWCNTs.

**Supplementary Note 2: UV-irradiation, XPS and TPD.** The azide modified MWCNT sample was prepared by uv-irradiation of the azide-soaked MWCNT. The plasma sample was prepared on carbon nanotube substrates in a nitrogen gas environment instead of sodium azide in order to eliminate contributions due to unreacted sodium azide in the XPS spectrum. Both the azide modified and plasma-reacted samples were compared to the pure azide (from sodium azide) and carbon nitride reference samples. The signals from both azide-modified and plasma-reacted samples were too weak to be observed without multiplying the signals by a factor of 104. The two prominent features in the green curve are the typical features from unreacted azide ion. The feature at 404 eV is probably from the species that is bonded to the carbon nanotube. The peaks are comparable in energy but not intensity ratio compared to the spectrum in red from sodium azide dispersed on a MWCNT substrate. The higher intensity of the line near 400 eV that is also shifted slightly to lower binding energy compared to the higher binding energy azide N1s peak may indicate some PN formation. It would be difficult to assign this line to pyrrolic nitrogen since the TPD (temperature- programmed desorption) results (Supplementary Figure. 7) from this sample did not show a high temperature decomposition peak expected for pyrollic nitrogen. These features from the modified and reference samples are completely different from the N1s XPS spectrum from the plasma-reacted nitrogen sample which has primarily one feature at 398 eV consistent with that of the singly N-N bonded cg-PN phase. This result suggests that a PN phase can be obtained with both nitrogen and sodium azide as precursors.

**Supplementary Note 3: Particle size from Scherrer equation.** Based on the Scherrer equation [particle size, $d = k\lambda/\beta cos\theta$, where k is the shape factor, $\lambda$ is the x-ray wavelength, $\beta$ is the x-ray diffraction linewidth at half intensity in radians and $\theta$ is the Bragg angle in degrees] we calculated the particle sizes of the polymeric nitrogen structures from the x-ray diffraction data in Fig. 3 as shown in Supplementary Table. 1. With all three index peaks and shape factor 0.9, we get particle sizes ranging between 12 and 20 nm. However, as observed from the TEM images, the polymeric nitrogen nanoparticles are rod shaped instead of spherical in shape. Therefore, the particle sizes would range between 8 and 14 nm if we use a shape factor of 0.6. The lower end of these sizes can fit into the central tube with some bulging as evident in Fig. 4 b.

**Supplementary Table 1: Particle size from Scherrer equation.**

| | | | | | | |
|---|---|---|---|---|---|---|
| | | size ($\AA$) | | | | size ($\AA$) |
| K | 0.9 | 122.28 | | K | 0.6 | 81.52 |
| $\lambda$ ($\AA$) | 1.54 | | | $\lambda$ ($\AA$) | 1.54 | |
| $\beta$ (rad) | 0.014 | | | $\beta$ (rad) | 0.014 | |
| $\theta$ (deg) | 38.5 | | | $\theta$ (deg) | 38.5 | |
| | | | | | | |
| | | size ($\AA$) | | | | size ($\AA$) |
| K | 0.9 | 204.94 | | K | 0.6 | 136.63 |
| $\lambda$ ($\AA$) | 1.54 | | | $\lambda$ ($\AA$) | 1.54 | |
| $\beta$ (rad) | 0.007 | | | $\beta$ (rad) | 0.007 | |
| $\theta$ (deg) | 19 | | | $\theta$ (deg) | 19 | |
| | | | | | | |
| | | size ($\AA$) | | | | size ($\AA$) |
| K | 0.9 | 136.88 | | K | 0.6 | 91.25 |
| $\lambda$ ($\AA$) | 1.54 | | | $\lambda$ ($\AA$) | 1.54 | |
| $\beta$ (rad) | 0.012 | | | $\beta$ (rad) | 0.012 | |
| $\theta$ (deg) | 34 | | | $\theta$ (deg) | 34 | |
